# Acute hypophysitis and hypopituitarism in early syphilitic meningitis in a HIV-infected patient: a case report

**DOI:** 10.1186/1471-2334-13-481

**Published:** 2013-10-17

**Authors:** Christoph D Spinner, Sebastian Noe, Christiane Schwerdtfeger, Antonia Todorova, Jochen Gaa, Roland M Schmid, Dirk H Busch, Michael Neuenhahn

**Affiliations:** 1 Department of Medicine II, University Hospital Klinikum rechts der Isar, Ismaningerstr. 22, 81675 Munich, Germany; 2 Department of Dermatology and Allergy, University Hospital Klinikum rechts der Isar, Biedersteiner Str. 29, 80802 Munich, Germany; 3 Department of Radiology, University Hospital Klinikum rechts der Isar, Ismaningerstr. 22, 81675 Munich, Germany; 4 Institute for Medical Microbiology, Immunology and Hygiene, Technische Universitaet Muenchen (TUM), Trogerstr. 30, 81675 Munich, Germany; 5 Interdisciplinary HIV Centre (IZAR), University Hospital Klinikum rechts der Isar, Ismaningerstr. 22, 81675 Munich, Germany; 6 German Center for Infection Research (DZIF), Munich, Germany

**Keywords:** HIV, Syphilis, Hypopituitarism, Hypophysitis

## Abstract

**Background:**

Sexually transmitted diseases and most notably syphilis-infections are rising amongst men who have sex with men. In HIV-co-infected patients, an accelerated clinical course of syphilis neurological involvement is known.

**Case presentation:**

A 46 year old HIV-positive male patient came in to our emergency department in the late evening with acute fever, rapidly progressive cephalgia and photophobia. Palmar skin efflorescence was evocative of an active syphilis infection. A reactive Treponema pallidum particle agglutination (TPPA) assay with positive *Treponema pallidum*-specific IgG/IgM immunofluorescence as well as a highly reactive Veneral diseases research laboratory (VDRL) test confirmed the diagnosis. Liquor pleocytosis, liquor protein elevation and a highly positive VDRL test in cerebrospinal fluid (CSF) were interpreted in context of the clinical symptoms as neurosyphilitic manifestations within an early syphilis infection (stage II). Cranial nuclear magnetic resonance scans of the sella turcica, which were performed due to low thyroidea stimulation hormone (TSH) and thyroxin levels, showed signs of hypophysitis such as pituitary gland enlargement and inhomogeneous contrast enhancement. Advanced endocrine laboratory testing revealed hypopituitarism. Fourteen days of intravenous ceftriaxone treatment and levothyroxine- and hydrocortisone-substitution led to complete disappearance of all clinical symptoms. Two months later, nuclear magnetic resonance scan showed normal pituitary size and that the syphilis serology had normalized.

**Conclusion:**

We report to the best of our knowledge the first case of a HIV-positive patient with acute hypophysitis and hypopituarism due to early neurosyphilis infection. Ceftriaxone treatment and levothyroxine- and hydrocortisone-substitution led to the disappearance of all clinical symptoms. We strongly recommend to exclude syphilis infection in every clinical situation unclear in HIV-patients, especially when additional risk factors are known.

## Background

Sexually transmitted diseases (STD) and in particular syphilis infections, caused by the spirochete *Treponema pallidum* (subspecies pallidum), are on the rise since the beginning of this century [1-3]. Men who have sex with men (MSM) are by far the most affected group in the western world and Human immunodeficiency virus (HIV) co-infected MSM have been described to show an accelerated and more complicated course of syphilis infections with neurological involvement [4-7]. Notably, cerebrospinal fluid (CSF) and neurological abnormalities (e.g. meningitis) correlate with low (<=350 cells/μl) CD4 cells counts in HIV seropositive individuals [8,9]. Syphilis infection has also been linked to a specific pituitary inflammation, named granulomatous hypophysitis, in analogy to other granulomatous infectious etiologies [10-13]. There is, however, one case report of pituitary gland gumma in a HIV-negative congenital syphilis case [14]. We here describe to our best knowledge the first clinical case of acute hypophysitis during an early syphilis infection in an adult HIV-positive patient. Though a rare disorder, physicians should be aware of the possibility of pituitary inflammation secondary to syphilis, since a potential secondary adrenal insufficiency may develop, which can be lethal if unrecognized and not treated adequately and immediately.

## Case presentation

**Figure 1:**
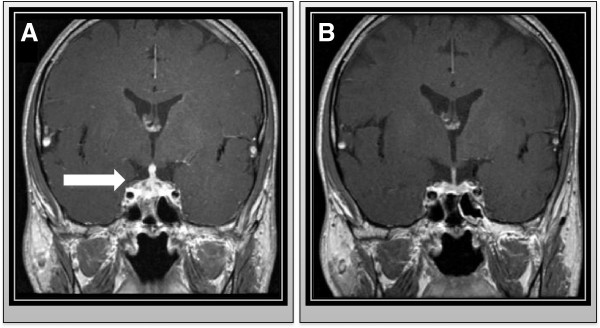
**NMR scan of enlarged pituitary at diagnosis and after treatment. A**. Contrast-enhanced coronal T1-weighted nuclear magnetic resonance (NMR) scan of the sella turcica showing pituitary and pituitary stalk enlargement with inhomogeneous contrast enhancement. **B**. Contrast-enhanced coronal T1-weighted nuclear magnetic resonance (NMR) scan of the sella turcica two months after hypophysitis demonstrates normal pituitary size.

We here report a case of acute hypophysitis and consecutive hypopituitarism due to an active neurosyphilis infection in a 46-year old HIV-positive MSM patient. The patient had rapidly progressive cephalgia for two weeks and rapid symptom progression (accentuated in the evening) during the last two days before hospitalization. On the day of admission, he arrived at our emergency department with acute fever (39°C) and pronounced photophobia. Beside the combined antiretroviral therapy with Abacavir, Lamivudin and Efavirenz no other medication was taken upon first presentation. The HIV load had been below detection level (< 20 cps/ml) at his last outpatient visit two months ago and a recent CD4-count was 507 cells/μl (27%, Ratio CD4/CD8-ratio 0.7). Beside the known HIV-infection, the STD history was unremarkable. Six months before the actual admission, a screening test for syphilis co-infection using the Treponema pallidum particle agglutination (TPPA) assay had been negative. Drug abuse was denied and other pre-existing medical conditions were not known. The HIV diagnosis in this patient was first made five years ago due to pneumocystis pneumonia, thrush esophagitis and buccal mucosa Kaposi’s sarcoma. CDC stage at first diagnosis was C3 and CD4-nadir was 93 cells/μl. (8%, CD4/CD8-ratio 0.1). The patient was hospitalized from the emergency department and on the assumption of acute meningitis was immediately administered a calculated antimicrobial therapy with intravenous Ceftriaxone (4 g per day on day 1, followed by 2 g per day), Ampicillin (6 × 2 g per day) and Aciclovir (3 × 10 mg/kg body weight per day). An emergency cranial computed tomography ruled out signs of intracranial pressure. Lumbar puncture showed mild pleocytosis (10 cells/μl) and high protein levels (1040 mg/l). The liquor/serum-albumin-quotient was elevated, indicating blood-CSF-barrier disturbance (13 × 10^-3^). Initial laboratory diagnostics revealed hyponatraemia (130 mmol/l), mild C-reactive protein elevation (2.1 mg/dl) and abnormal liver function tests (ALT 68 U/l, AST 85 U/l). Notable TSH suppression (0,15 μIU/ml) and low free thyroxine levels (fT4) (0,4 ng/dl) in the routine screening laboratory test upon emergency admission led to cranial nuclear magnetic resonance (NMR)-imaging of the sella turcica. This revealed pituitary stalk enlargement with inhomogeneous contrast enhancement (Figure 1A). Advanced endocrine laboratory analysis was performed five days after admission and showed hypopituitarism with low levels for TSH (0.15 μIU/ml), fT3 (1,6 pg/ml), fT4 (0,3 ng/ml), luteinising-hormone (LH) (< 0,1 IU/l), testosterone (< 0,1 IU/l), basal cortisone (0,2 μg/dl) and insulin-like growth factor (IGF-1) (74 ng/ml). The patient refused a stereotactic biopsy and histopathological assessment to rule out malignancy of the pituitary enlargement.

Subsequent microbiological and viral analysis by nucleic acid amplification ruled out infection of the central nervous system through Mycobacterium tuberculosis, Herpes simplex-, Cytomegalo-, Epstein-Barr-, Varicella- or Enterovirus. Aerobic and anaerobic liquor cultures were sterile. HIV PCR from peripheral blood showed low-level viremia (47 copies/ml), but was tested negative in the cerebrospinal fluid. Upon clinical re-examination erythematous maculae with distinct edges were detectable on the palms and soles. According to the patient the maculae had been appeared three weeks ago. Consistent with these skin manifestations, a disseminated early syphilis infection (stage II) was assumed. Syphilis infection was serologically confirmed by reactive TPPA (1:10240) and a positive Treponema pallidum-specific IgG immunofluorescence (IgG-FTA abs) test. The detection of Treponema pallidum-specific IgM (IgM-FTA abs and immunoblot) together with high titers in the Venereal Disease Research Laboratory (VDRL) micro flocculation test (1:512) in patient serum indicated high activity of the syphilis infection. Because of the meningitis symptoms and the known accelerated clinical course of syphilis in HIV-patients specific liquor diagnostics were performed. Syphilis-specific liquor serology showed a positive TPPA (1:64) and a highly positive VDRL test (1:512) in CSF. However, the TPPA-liquor/serum index (ITpA-index) was normal (1.15) and a retrospectively performed Treponema pallidum-specific NAT in CSF was negative. The empirically started Ceftriaxone therapy (2 g per day) led to rapid resolution of the fever within the first day of treatment and the resolution of cephalgia and photophobia within the first two days. Neurosyphilis treatment was continued for a total course of fourteen days. After exclusion of cerebral Listeria and HSV infection, the treatment with Aciclovir and Ampicillin was stopped. Hormone substitution therapy with levothyroxine (75 μg per day) and hydrocortisone substitution (50 mg per day) was started for the hypopituitarism and the laboratory values normalized within days. Under ceftriaxone therapy and levothyroxine and hydrocortisone substitution all clinical symptoms completely cleared. A NMR scan of the sella turcica two months later showed a normal pituitary size (Figure 1B) and the patient remained asymptomatic. Hormone substitution was reduced gradually and then completely stopped. Hormone laboratory results (TSH, fT3, fT4, cortisone basal level) subsequently remained consistently normal. 6 months after hospitalization, syphilis serology confirmed a significantly reduced TPPA titer (1:320), negative Treponema pallidum-specific IgM immunofluorescence and a normalized VDRL test (<1:2) in the patient’s serum, indicating successful antibiotic treatment.

## Conclusion

We report to the best of our knowledge the first case of a HIV-positive MSM patient with acute hypophysitis and hypopituarism due to an active syphilis infection. In the context of the clinical symptoms (meningitis and disseminated cutaneous exanthema) and a negative syphilis screening history (only six months before hospitalization) we interpreted the clinical picture as a confirmed early secondary syphilis (according to ECDC definitions) [1]. The high activity of the infection was illustrated by positive *Treponema pallidum*-specific serum IgMs and high VDRL test serum titers (1:512). In order to treat the presumably syphilitic lesions in the pituitary gland and the neurological symptoms sufficiently, we decided to continue intravenous antibiotic treatment for 14 days according to national treatment guidelines [4]. CSF abnormalities with blood-CSF-barrier disturbance, CSF pleocytosis, high liquor protein levels (total protein 1.040,0 mg/l) and in particular a very high CSF VDRL test reactivity (1:512) were in line with early neurosyphilis in accordance to CDC guidelines [8]. HIV-independent *Treponema pallidum* invasion of the CSF in patients with early syphilis is known in approximately one-third of all cases [10]. Interestingly the number of neurosyphilis cases in early syphilis-infected patients increased in the HIV era rapidly [2,3,15]. This might be due to impaired syphilis infection control after CNS involvement due to compromised host immune activity as a result of HIV-infection [5-7,10]. Especially HIV-patients with a CD4-count < 350 cells/μl seem to be under a higher risk of developing cerebrospinal fluid (CSF) and neurological abnormalities (e.g. meningitis) [9]. However, in this case, symptomatic bacterial replication in the central nervous system could not be formally proven according to national diagnostic criteria, because the ITpA index was not elevated [4,11-13]. A retrospectively performed Treponema pallidum-specific NAT from CSF was also negative, but a recent meta-analysis reported low sensitivity (47%) in retrospective CSF PCR samples from patients with neurosyphilis [14,16]. In any case, the complete neurological, endocrinological and serological recovery after ceftriaxone treatment count as the strongest argument for *Treponema pallidum*-caused hypophysitis and consecutive hypopituitarism.

We strongly recommend to rule out syphilis in every uncertain clinical situation in HIV-patients, especially when additional risk factors (MSM or others) are known. Syphilis-specific CSF diagnostic should be performed whenever any additional signs of neurosyphilis occur. A CSF VDRL test could be helpful to diagnose neurosyphilis when other signs of neurosyphilis are missing.

### Consent statement

Written informed consent was obtained from the patient for publication of this report and accompanying images. A copy of written consent is available for review by the Editor of this journal.

## Competing interests

We declare for all authors that there is no competing interests.

## Authors’ contributions

AT was responsible for the dermatological diagnostics, SN for the endocrinological diagnostics and therapy and CSp for the infectious counselling as well as the complete clinical care and treatment of the patient. JG cared for radiology diagnostics, especially the NMR and cranial CT scan. MN contributed for microbiological laboratory and serological diagnostics. CS and MN wrote the manuscript with equal contributions. In addition, CSc was relevantly involved in data interpretation, additional retrospective liquor analysis and manuscript composition. RS and DB supervised clinical and laboratory diagnostics and therapy, as well as manuscript preparation. We declare that all authors contributed material relevant to the manuscript and have read and approved the final version.

## Pre-publication history

The pre-publication history for this paper can be accessed here:

http://www.biomedcentral.com/1471-2334/13/481/prepub
